# A postpartum functional assessment tool for women based on the international classification of functioning, disability and health

**DOI:** 10.1186/s12905-024-02880-z

**Published:** 2024-01-06

**Authors:** Ying Zhao, Meng Yuan, Jie Wu, Zhao Wang, Fan Jia, Lili Ma, Yang Yang, Jingjie Zhou, Ming Zhang

**Affiliations:** 1 The Affiliated Xuzhou Rehabilitation Hospital of Xuzhou Medical University, Xuzhou, 221003 China; 2 The Xuzhou Clinical College of Xuzhou Medical University, Xuzhou Central Hospital, Xuzhou, 221009 China

**Keywords:** Postpartum, Function, International classification of functioning, disability and health, Expert consensus

## Abstract

**Background:**

Postpartum dysfunctions and complications can occur in women. However, functional assessment should be conducted to make treatment plans before any intervention is implemented. In this context, the International Classification of Functioning, Disability and Health (ICF) may be a useful tool for women postpartum to document functional data and set rehabilitation goals. The purpose of this study was to determine the corresponding domains that should be considered in the evaluation of women’s postpartum functioning based on the International Classification of Functioning, Disability and Health (ICF) model using the Delphi method.

**Methods:**

Fifteen domestic experts were invited to conduct two rounds of expert consensus survey on the ICF-based postpartum functional assessment category pool obtained through literature retrieval, clinical investigation, and reference to relevant literature. The sample was medical staff with professional knowledge of women’s health. The opinions of experts were summarized, and the positive coefficient, authority coefficient and coordination degree of experts were calculated.

**Results:**

A total of 15 domestic experts participated in this expert consensus. Through two rounds of a questionnaire survey, 69 items were finally selected to form the ICF-based postpartum functional assessment tool for women. The items included 32 items of body function, 12 items of body structure, 17 items of activity and participation, and 8 items of environmental factors. In addition, we identified 8 items of personal factors. The expert positive coefficients of the two rounds of expert consensus were both 100%, the authority coefficient was 0.789, and the coefficient of variation was between 0.09 to 0.31.

**Conclusion:**

A postpartum functional assessment tool for women based on the ICF model was constructed based on the Delphi method, which can provide more comprehensive health management and life intervention for postpartum women.

**Trial registration:**

The Registration number of the Chinese Clinical Trial Registry is ChiCTR2200066163, 25/11/2022.

**Supplementary Information:**

The online version contains supplementary material available at 10.1186/s12905-024-02880-z.

## Background

According to the World Health Organization (WHO), the postpartum period is a critical transition period for the mother, the baby, and the family at the physical, psychological, and social levels [1]. Thus, in addition to intrinsic biological factors, environmental and personal factors are determinants of postpartum maintenance or recovery of health, function, and quality of life in women. Every year, there are approximately 15 million parturient women in China. The frequency of delivery is relatively high. However, the postpartum rehabilitation is still vaguely defined and under-explored. Postpartum recovery is a complex, multidimensional structure with cultural variables. Studies have shown that women have postpartum impairments in physical functioning, mental health, and sleep as well as deficits in social support [2–6]. However, clinical postnatal screening and rehabilitation are still limited to the examination of the pelvic floor, rectus abdominis and breast. The treatment and assessment methods are relatively simple. There is still a lack of a developed and validated tool to comprehensively assess postnatal recovery after hospital discharge.

To support the biopsychosocial approach, the World Health Organization (WHO) published the International Classification of Functioning, Disability and Health (ICF) in 2001 [7]. ICF complements the International Classification of Diseases (ICD) [8] by providing uniform and standardized terminology to describe individual functioning and contextual factors that affect health. The function is an umbrella term that includes body function (b) and body structure (s) as well as activity and participation (d) sections. Background factors include environmental factors (e) as well as unclassified personal factors, which can be either facilitators or barriers to an individual’s health status [9]. In addition, ICF can integrate information on impairments and limitations with other functional elements important to learning such as participation, to improve the description of health status and impairment, identify the critical role of contextual factors, and provide a basis for goal setting by supporting the integration of assessment information from different sources, settings, and perspectives [10]. At present, ICF has been widely promoted and applied worldwide with good reliability, validity, and feasibility [11].

The WHO has been committed to using ICF as a universal assessment tool to evaluate health and function and to promote its application in the world. At present, it has developed a comprehensive core set of 33 diseases and formulated a brief core set for specific diseases according to the actual needs of clinical work [12–15]. Rehabilitation focuses on function and human response to disease, disability, or limitation rather than on specific pathological conditions, and the overall principles of rehabilitation are theoretically consistent with ICF. Based on the ICF model, this study aims to determine the postpartum functional assessment tool for women through the Delphi method [16], which may provide some guidance for clinical postnatal examination, assessment and rehabilitation treatment.

## Methods

### Selection of experts

In this study, Experts from the Pelvic Floor Rehabilitation Group of the Rehabilitation Treatment Special Committee of the Chinese Association of Rehabilitation Medicine were invited to participate in the survey. Inclusion criteria: (1) engaged in gynecology/obstetrics, postpartum rehabilitation; (2) associate senior professional title or engaged in gynecology/obstetrics, postpartum rehabilitation work for 5 years or more; (3) working in tertiary hospitals or above; (4) bachelor’s degree or above; and (5) willingness to accept the study consultation. According to the inclusion criteria, 15 experts were selected to participate in this expert consensus, the city of experts was not selected apriori. We counted the region where each expert was located, the 15 experts were from 11 cities in 10 provinces such as Jiangsu, Tianjin, Shenzhen, Liaoning, Jilin, Yunnan, Hainan, Zhejiang, Heilongjiang, and Shanghai. Two rounds of expert consultation were conducted in February and March 2023.

### Screening and establishment of the ICF item pool and development of the initial questionnaire

Based on a) literature retrieval, b) clinical investigation, and c) reference to relevant literature [17], this study initially constructed the item pool of postpartum functional assessment for women based on the ICF model (A total of 83items, detailed data are shown in Supplementary Table 1).

- Literature retrieval: Using the method of evidence-based medicine, two researchers who were familiar with ICF and engaged in postpartum rehabilitation independently conducted literature screening and concept extraction. PubMed, CNKI, WanFang and other databases were searched for the common related dysfunctions and influencing factors in the clinical examination, rehabilitation treatment and evaluation of postpartum women in the past five years. The concepts contained in the indicators were extracted, their contents were defined and analyzed, and linked to the ICF. If there were different opinions, a third person was consulted. Chinese search terms: (postpartum women OR puerpera) AND (function OR structure) AND (rehabilitation OR treatment). English search terms: postpartum AND (functioning OR structure) AND (rehabilitation OR therapy). Inclusion criteria: (1) postpartum women; (2) the theme of the study was the commonly related dysfunctions and influencing factors of postpartum women in clinical examination, rehabilitation treatment and evaluation; and (3) the research design was clinical randomized controlled trial, observational study, cross-sectional study, and qualitative study. Exclusion criteria: (1) related dysfunction occurred during the prenatal examination; (2) animal experiment, clinical phase II experiment, basic research, case study; (3) unable to access the full text; and (4) unpublished papers, such as dissertations (master’s degree, doctor’s degree), conference journals, reviews, etc. The concept of indicators was extracted and linked with ICF, and the questionnaire form of the WHO ICF checklist was used to develop a postpartum functional assessment scale. A total of 73 ICF items that may be related to postpartum dysfunction in women were included.
- Clinical investigation: To test whether the items obtained from the literature retrieval are applicable to clinical examinations, the postpartum function assessment form obtained from Literature retrieval was used for clinical investigation. In the clinical investigation, 300 patients who visited the postpartum rehabilitation department of our hospital were selected, and 30 medical staff who had long-term working experience in the main branch and Xincheng branch of Xuzhou Central Hospital were selected as the medical survey objects, and paper questionnaires were distributed. Patient inclusion criteria: (1) age 20–45 years old; (2) 42 days to 6 months postpartum; (3) no serious diseases in prenatal examination; and (4) good compliance, can actively cooperate to complete the questionnaire and the corresponding examination. Patient exclusion criteria: (1) severe underlying diseases before delivery, affecting postpartum health status; and (2) patients with a history of epilepsy, mental illness or other neurological diseases affecting cognition, who cannot cooperate with the relevant assessment. Medical staff inclusion criteria: (1) working in the department of postpartum rehabilitation/ obstetrics/ gynecology for more than 3 years; and (2) consent to participate in this study. Medical staff exclusion criteria: (1) did not complete the questionnaire due to personal reasons; and (2) have a long-term leave or not working in the relevant position in the past three months. Among the 30 medical staff, 18 were obstetric staff, 7 medical staff of postpartum rehabilitation department, 5 gynecological medical staff. There were 8 chief physicians, 2 associate chief physicians and 8 attending physicians. There were 4 supervising postpartum rehabilitation therapist and 1 postpartum rehabilitation therapist. There were 2 chief nurses, 3 supervisor nurses, and 2 nurse practitioners. A total of 300 patients were investigated and evaluated clinically, and the frequency of each ICF item was counted. All 73 items were involved in postpartum dysfunction. In order to select the common dysfunction problems in clinical postpartum women, the item with dysfunction frequency rank sum ≥ 30% was used as the first ICF item pool for postpartum functional assessment. Questionnaires were distributed to medical staff to count the ICF items that medical staff thought were closely related to women’s postpartum function, and more than 50% of the items were used as the second stage ICF item pool. The results of the two periods were integrated to construct the core category set of postpartum functional assessment for women based on ICF (See Supplementary Table 2 notes for specific integration examples). Finally, ICF category pool obtained from the patient survey and clinical survey was used for expert consultation. The final 63 items were common dysfunction problems in women (The detailed data are shown in Supplementary Table 2). Its significance is to provide a certain reference for expert consensus. If experts have different opinions on an item, we can refer to whether the item is a common dysfunction problem for women, so as to decide whether the item should be retained.
- Reference to relevant literature: Bulhões et al. [17] selected 45 physical therapists and finally determined 53 ICF categories and 9 personal factor items through three rounds of letter consultation. In order to ensure that the items in the letter consultation were more comprehensive, we referred to the Brazilian consensus, and included the items that were not covered by the Brazilian consensus but may be related to postpartum dysfunction of Chinese women. The included items were: “b7305 Power of muscles of the trunk”, “b760 Control of voluntary movement functions”, “b730 Muscle power functions”, “d760 Family relationships”, “d770 Intimate relationships”, “e120 Products and technology for personal indoor and outdoor mobility and transportation”, “e155 Design, construction, and building products and technology of buildings for private use”, “e460 Societal attitudes”, “e540 Transportation services, systems, and policies”, “e5800 Health services”. A total of 10 items were included, with the addition of the previous 73 items, a total of 83 items were obtained.

Based on the items obtained from a) literature retrieval, b) clinical investigation, and c) reference to relevant literature, we began to develop the questionnaire and divided the content into 3 parts: (1) explanation of the questionnaire: sending a letter to the experts, explaining the purpose and significance of the study, the background information of the study and the instructions of filling the form; (2) content of the questionnaire: Likert 5-point scoring method was used to evaluate the importance of postpartum function assessment items. The values of “very important”, “important”, “moderately important”, “not very important” and “not important” were respectively assigned 5 points, 4 points, 3 points, 2 points and 1 point [18]. Open-ended suggestions were added after the items to collect items that experts considered important but were not included in the questionnaire; and (3) basic information and authority degree of experts: including general information such as gender, age, educational background, major, professional title, working years, etc. In addition, at the end of each part of the items, we append a questionnaire on the judgment basis and familiarity of experts to the consultation items to calculate and judge the authority of experts.

### Delphi expert consensus process

The Delphi method was used to preliminarily determine the items included in ICF functional assessment tool for women postpartum based on two rounds of expert consensus, and an online survey was used to send questionnaires to experts. An anonymous statistical method was adopted, and experts were not allowed to communicate with each other with no horizontal contact and could only communicate with investigators [19]. After two rounds of expert consultation, the items in the ICF item pool were screened and improved according to the survey results. Finally, the ICF item pool was summarized based on consistent expert opinions. Content validity was assessed in two phases which are development phase and the judgment quantification phase. The purpose of the current study was limited to the latter stage, which was to judge the items of the existing ICF item pool, so a two-round Delphi survey was considered adequate [20]. Questionnaires were sent out in the form of emails, and the time between the two rounds was about 4 weeks. For each round, experts were given 2 weeks to respond [21]. The suggestions for the selection of ICF items include: (1) it is recommended to keep the common items of postpartum dysfunction in women; (2) for items with similar dimensions, it is suggested to keep the most representative items; and (3) it is suggested to delete the items that cannot be quantitatively evaluated clinically.

### Statistical methods

A database was established, and SPSS27.0 software was used for data analysis. The positive coefficient, authority degree, importance of items and coordination degree of experts were calculated. The calculation method is as follows: (1) expert positive coefficient: expressed as questionnaire recovery rate. Questionnaire recovery rate = (number of valid questionnaires /number of questionnaires issued) *100%; (2) expert authority (Cr) was calculated by the judgment coefficient (Ca) of the item content and the degree of familiarity (Cs) of the experts. Cr= (Ca + Cs) /2. It is generally believed that the authority coefficient of experts ≥ 0.70 has better authority [22]. The degree of familiarity was divided into five levels: “very familiar”, “relatively familiar”, “generally familiar”, “less familiar”, and “not familiar”. The judgment basis was divided into 4 aspects: theoretical analysis, practical experience, reference to domestic and foreign materials, and intuitive feeling. According to the degree of influence of the judgment basis on the questionnaire survey, experts choose from these three grades: large, medium, and small [23]; (3) importance of items: The Likert five-point scoring method was used to judge, the higher the score, the higher the importance of the item; and (4) the degree of expert coordination is expressed by the coefficient of variation (CV) of each item, CV = σ/M, where σ represents the standard deviation of the item and M represents the average of the item. The smaller the CV value, the higher the consistency of the expert’s judgment [17].

## Results

### Basic information of experts

The basic information of the 15 experts is shown in Table 1. Among the 15 experts, there were 3 males and 12 females, the experts in this study had a high degree of enthusiasm, and the recovery rate of the two rounds of questionnaire surveys was 100%. The professional degree and regional representativeness of the experts are also high, and 15 experts are distributed in 11 cities of 10 provinces. All experts have associate senior titles or above, have working experience is more than 10 years, and have a bachelor’s degree or above. Among them, 8 experts had more than 20 years of working experience, and 5 experts had more than 30 years of working experience, which could effectively control the content validity of the assessment tool. The professional direction of the experts covers gynecology, obstetrics, and postpartum rehabilitation, with postpartum rehabilitation as the main part. Therefore, the clinical rehabilitation needs of women can be understood from the perspective of rehabilitation treatment to guide rehabilitation treatment and evaluate the efficacy of rehabilitation treatment.

**Table 1:** Basic information of 15 experts

Variables	Frequency of survey(persons)	Percentage(%)
Gender		
Male	3	20%
Female	12	80%
Age(years)		
< 40	1	6.7%
40–49	5	33.3%
≥ 50	9	60%
Years of work(years)		
10–19	2	13.3%
20–29	8	53.3%
≥ 30	5	33.3%
Educational background		
Undergraduate	6	40%
Master	8	53.3%
PhD	1	6.7%
Professional title		
Associate professor	5	33.3%
Professor	10	66.7%
specialty		
Gynecology	2	13.3%
Obstetrics	2	13.3%
postnatal Rehabilitation Department	9	60%
Gynecology, Rehabilitation	2	13.3%

### Expert evaluation indicators

#### Expert positive coefficient

The positive coefficient of experts reflects the degree of attention of experts to the study. In this study, the questionnaire recovery rate was used to calculate the positive degree of experts. Fifteen questionnaires were distributed and 15 were recovered, and the positive coefficient of experts was 100%.

#### Expert authority index

The average familiarity degree of experts in each part of the items was 0.67, the average judgment basis was 0.91 and the final authority index was 0.789. Among them, the authority index of Body function was 0.803, the authority index of Body structure was 0.803, the authority index of Activities and participation was 0.83 and the authority index of Environmental factors was 0.723, indicating that the expert consensus in this study had good authority.

#### Expert coordination coefficient

The coordination coefficient represents the degree of consistency in the evaluation of the item, we use the coefficient of variation to express it. The smaller the coordination coefficient is, the more unified the opinions of the experts are. A general coefficient of variation < 0.3 represents a high degree of agreement among experts [18]. In this study, the coefficient of variation of the first round of the expert consensus survey was from 0.09 to 0.66, and that of the second round was from 0.09 to 0.31 indicating a high degree of consistency of experts in the second round of the survey.

### Determine the entry

#### The first round of survey

In the first round of the survey, 15 questionnaires were distributed and 15 were recovered with a 100% expert positive coefficient. Among the 83 ICF items, 14 items were deleted because their mean importance was less than 3 points. 16 ICF entries had mean importance scores between 3.0 to 3.5, and the rest had mean importance scores above 3.5. Among the items whose mean importance score was greater than 3.5, 21 items had a coefficient of variation greater than 0.3. The detailed data are shown in Table 2.

**Table 2:** First round survey data (*n* = 15)

ICF category	The frequency of each score was evaluated for importance(persons)					Mean value	Coefficient of variation
	very important (5 points)	important (4 points)	moderately important (3 points)	not very important (2 points)	not important (1 point)		
Body functions component							
b1300 Energy functions	6	8	1	0	0	4.33	0.14
b134 Sleep functions	8	6	1	0	0	4.47	0.14
b144 Memory functions	4	4	6	0	1	3.60	0.30
b152 Emotional functions	9	6	0	0	0	4.60	0.11
b280 Sensation of pain	8	6	1	0	0	4.47	0.14
*b28011 Chest pain	3	4	6	1	1	3.47^b^	0.33
b28012 Stomach and abdomen pain	6	4	2	2	1	3.80	0.35
b28013 Back pain	5	7	3	0	0	4.13	0.18
*b28014 Upper extremity pain	2	4	4	1	4	2.93^a^	0.49
b28015 Lower extremity pain	4	6	4	0	1	3.80	0.28
*b2802 Pain in multiple parts of the body	4	3	6	0	2	3.47^b^	0.38
*b415 Blood vessel functions	2	2	6	2	2	2.87^a^	0.45
b420 Blood pressure function	4	6	4	0	1	3.80	0.28
*b430 Blood system function	3	4	2	1	5	2.93^a^	0.55
b440 Respiration functions	4	6	3	1	1	3.60	0.33
b4552 Easy fatigue	3	7	3	0	2	3.60	0.35
b460 Sensations related to cardiovascular and respiratory function	4	4	5	1	1	3.60	0.33
b515 Digestive functions	4	4	6	0	1	3.67	0.30
b525 Defecation functions	6	8	1	0	0	4.33	0.14
b535 Sensations associated with the digestive system	2	6	6	1	0	3.60	0.23
b555 Endocrine gland function	4	4	5	0	2	3.53	0.37
b620 Urination functions	10	4	1	0	0	4.60	0.14
b6202 Urinary continence	10	5	0	0	0	4.67	0.11
b640 Sexual functions	7	7	1	0	0	4.40	0.14
b660 Reproductive functions	7	6	1	0	1	4.20	0.26
b6603 Lactation	7	5	1	0	2	4.00	0.34
b670 Sensations related to reproduction and reproductive function	6	7	0	0	2	4.00	0.33
b730 Muscle power functions	9	5	1	0	0	4.53	0.14
b7305 Power of muscles of the trunk	6	4	4	2	1	3.93	0.30
b735 Muscle tone functions	8	5	0	0	1	4.13	0.33
b740 Muscular endurance function	7	8	0	0	0	4.47	0.12
*b760 Control of voluntary movement functions	3	5	4	1	2	3.40^b^	0.38
*b770 Gait	4	4	4	1	2	3.47^b^	0.39
b780 Sensations related to muscle and motor function	5	6	2	0	2	3.80	0.35
*b820 Repair functions of the skin	3	7	1	1	3	3.40^b^	0.43
Body structures component							
*s410 Structure of cardiovascular system	1	5	4	2	3	2.73^a^	0.59
*s430 Structure of respiratory system	4	3	5	2	1	3.47^b^	0.36
s610 Structure of urinary system	7	7	1	0	0	4.40	0.14
s620 Structure of pelvic floor	12	3	0	0	0	4.80	0.09
s630 Structure of reproductive system	9	5	1	0	0	4.53	0.14
s6300 Ovary	4	4	5	1	1	3.60	0.33
s6301 Structure of uterus	9	5	1	0	0	4.53	0.14
s6302 Breast and nipple	6	6	2	0	1	4.06	0.27
s6303 Structure of vagina and external genitalia	10	3	2	0	0	4.53	0.16
*s730 Structure of upper extremity	2	2	7	2	2	2.89^a^	0.50
*s7302 Structure of hand	1	5	4	2	3	2.93^a^	0.49
s740 Structure of pelvic region	10	5	0	0	0	4.67	0.10
*s750 Structure of lower extremity	4	4	4	2	1	3.39^b^	0.37
s760 Structure of trunk	7	5	2	0	1	4.13	0.27
s7601 Muscles of trunk	7	5	2	0	1	4.13	0.27
Activities and participation component							
d230 Carrying out daily routine	7	4	1	2	1	3.93	0.34
d240 Handling stress and other psychological demands	8	5	1	1	0	4.33	0.21
d415 Maintaining a body position	5	6	3	1	0	4.00	0.23
d450 Walking	6	4	3	1	1	3.87	0.32
d570 Looking after one’s health	3	7	3	1	1	3.67	0.30
*d5700 Ensuring one’s physical comfort	5	1	5	1	3	3.27^b^	0.47
d5701 Control diet and regulate body	4	4	5	1	1	3.60	0.33
d640 Doing housework	4	6	2	2	1	3.67	0.34
d660 Assisting others	3	5	5	2	0	3.60	0.27
*d6600 Assisting others with self-care	2	5	7	0	1	3.46^b^	0.29
*d760 Family relationships	6	3	1	1	3	3.47^b^	0.47
d770 Intimate relationships	5	8	1	0	1	4.07	0.25
d7702 Sexual relationships	7	8	0	0	0	4.47	0.12
d850 Remunerative employment	4	9	1	0	1	4.00	0.25
*d920 Recreation and leisure	3	6	2	2	2	3.40^b^	0.40
d9201 Sports	3	5	5	1	1	3.53	0.32
*d9205 Socializing	2	7	3	1	1	3.47^b^	0.32
Environmental factors component							
*e115 Products and technology for personal use in daily living	2	5	3	0	5	2.93^a^	0.52
*e120 Products and technology for personal indoor and outdoor mobility and transportation	2	6	1	2	4	2.80^a^	0.70
*e155 Design, construction, and building products and technology of buildings for private use	0	5	2	0	8	2.26^a^	0.63
e310 Immediate family	6	4	2	1	2	3.73	0.39
*e315 Extended family	3	3	4	0	5	2.93^a^	0.54
*e320 Friends	3	5	3	1	3	3.26^b^	0.44
*e325 Acquaintances, peers, colleagues, neighbors, and community members	3	2	5	1	4	2.93^a^	0.51
*e355 Health professionals	7	2	1	2	3	3.33^b^	0.61
*e360 Other professionals	2	2	4	2	5	2.60^a^	0.56
e410 Individual attitudes of immediate family members	3	8	2	1	1	3.73	0.29
*e460 Societal attitudes	3	6	2	1	3	3.33^b^	0.43
*e540 Transportation services, systems, and policies	1	4	2	0	8	2.30^a^	0.66
*e575 General social support services, systems, and policies	5	2	5	1	2	3.47^b^	0.41
e580 Health services, systems, and policies	7	4	3	1	0	4.13	0.24
*e5800 Health services	3	3	3	2	4	2.93^a^	0.53
e590 Labor and employment services, systems, and policies	5	4	3	2	1	3.60	0.40

Note: *indicates that the item did not fulfill the criteria; ^a^ indicates that the mean importance of the item is less than 3.0; ^b^ indicates that the mean importance of items is between 3.0 to 3.5

(Corresponding to the first round of survey in the text)

#### The second round of survey

The questionnaire in the second round is composed of items whose mean importance is greater than 3.0 points in the results of the first round. The items whose mean importance is between 3.0 to 3.5 points or whose coefficient of variation is greater than 0.3 points will be highlighted as the content of this round of discussion. In the second round of the survey, 15 questionnaires were distributed, 15 questionnaires were recovered, and the positive coefficient of experts was 100%. Among the 69 ICF items, 67 entries had a mean importance score > 3.5 and a coefficient of variation < 0.3. In addition, one item d6600 “Assisting others with self-care” had a mean importance score of < 3.5 but > 3.0, and one item d415 “Maintaining a body position” had a mean importance score of > 3.5 but a coefficient of variation > 0.3. After discussion in the group and conjunction with the previous clinical survey (The detailed data are shown in Supplementary Table 2), d6600 and d415 were both common postpartum dysfunction questions in women, so we decided to retain these two items. Except for d415, the coefficients of variation of other items were all below 0.3, which can be considered that the experts have a high degree of consistency and a high degree of recognition for ICF items in the assessment tool and reflect the good content validity of this study. The detailed data are shown in Table 3.

**Table 3:** Second round survey data (*n* = 15)

ICF category	The frequency of each score was evaluated for importance(persons)						Mean value	Coefficient of variation
	very important (5 points)		important (4 points)	moderately important (3 points)	not very important (2 points)	not important (1 point)		
Body functions component								
b1300 Energy functions		9	5	1	0	0	4.53	0.14
b134 Sleep functions		11	4	0	0	0	4.73	0.10
b144 Memory functions		6	5	3	1	0	4.07	0.24
b152 Emotional functions		10	5	0	0	0	4.67	0.10
b280 Sensation of pain		10	3	2	0	0	4.53	0.16
b28011 Chest pain		4	5	6	0	0	3.87	0.22
b28012 Stomach and abdomen pain		3	9	3	0	0	4.00	0.16
b28013 Back pain		5	9	1	0	0	4.27	0.14
b28015 Lower extremity pain		4	8	3	0	0	4.07	0.17
b2802 Pain in multiple parts of the body		6	7	1	1	0	4.20	0.21
b420 Blood pressure function		4	8	3	0	0	4.07	0.17
b440 Respiration functions		9	4	3	0	0	4.47	0.17
b4552 Easy fatigue		6	5	4	0	0	4.13	0.20
b460 Sensations related to cardiovascular and respiratory function		7	7	1	0	0	4.40	0.14
b515 Digestive functions		4	5	6	0	0	3.87	0.22
b525 Defecation functions		8	6	1	0	0	4.47	0.14
b535 Sensations associated with the digestive system		5	7	3	0	0	4.13	0.18
b555 Endocrine gland function		4	10	1	0	0	4.20	0.13
b620 Urination functions		11	4	0	0	0	4.73	0.10
b6202 Urinary continence		11	4	0	0	0	4.73	0.10
b640 Sexual functions		8	5	2	0	0	4.40	0.17
b660 Reproductive functions		7	8	0	0	0	4.47	0.12
b6603 Lactation		9	5	1	0	0	4.53	0.14
b670 Sensations related to reproduction and reproductive function		8	6	1	0	0	4.47	0.14
b730 Muscle power functions		6	9	0	0	0	4.40	0.12
b7305 Power of muscles of the trunk		4	9	1	1	0	4.07	0.20
b735 Muscle tone functions		6	8	1	0	0	4.33	0.14
b740 Muscular endurance function		8	6	1	0	0	4.47	0.14
b760 Control of voluntary movement functions		6	6	2	1	0	4.13	0.22
b770 Gait		4	7	3	1	0	3.93	0.22
b780 Sensations related to muscle and motor function		7	7	0	1	0	4.33	0.19
b820 Repair functions of the skin		5	6	4	0	0	4.07	0.20
Body structures component								
s430 Structure of respiratory system		6	9	0	0	0	4.40	0.12
s610 Structure of urinary system		7	7	1	0	0	4.40	0.14
s620 Structure of pelvic floor		10	4	1	0	0	4.60	0.14
s630 Structure of reproductive system		8	6	1	0	0	4.33	0.14
s6300 Ovary		5	8	2	0	0	4.20	0.16
s6301 Structure of uterus		6	8	1	0	0	4.33	0.14
s6302 Breast and nipple		7	8	0	0	0	4.47	0.12
s6303 Structure of vagina and external genitalia		9	6	0	0	0	4.60	0.11
s740 Structure of pelvic region		12	3	0	0	0	4.80	0.09
s750 Structure of lower extremity		4	7	3	1	0	3.93	0.22
s760 Structure of trunk		7	7	1	0	0	4.40	0.14
s7601 Muscles of trunk		7	7	1	0	0	4.40	0.14
Activities and participation component								
d230 Carrying out daily routine		6	6	3	0	0	4.20	0.18
d240 Handling stress and other psychological demands		5	9	1	0	0	4.27	0.14
d415 Maintaining a body position		5	6	2	1	1	3.87	0.31
d450 Walking		6	4	5	0	0	4.07	0.22
d570 Looking after one’s health		6	5	3	1	0	4.07	0.24
d5700 Ensuring one’s physical comfort		3	6	5	0	0	3.73	0.24
d5701 Control diet and regulate body		4	7	3	1	0	3.93	0.22
d640 Doing housework		1	9	5	0	0	3.73	0.16
d660 Assisting others		3	8	4	0	0	3.93	0.18
*d6600 Assisting others with self-care		2	5	7	0	1	3.47^b^	0.19
d760 Family relationships		6	8	1	0	0	4.33	0.14
d770 Intimate relationships		8	7	0	0	0	4.53	0.11
d7702 Sexual relationships		5	8	2	0	0	4.20	0.16
d850 Remunerative employment		5	10	0	0	0	4.33	0.11
d920 Recreation and leisure		2	11	2	0	0	4.00	0.14
d9201 Sports		1	11	2	1	0	3.80	0.18
d9205 Socializing		6	8	1	0	0	4.20	0.18
Environmental factors component								
e310 Immediate family		7	8	0	0	0	4.47	0.12
e320 Friends		4	7	3	1	0	3.93	0.22
e355 Health professionals		6	6	2	1	0	4.13	0.22
e410 Individual attitudes of immediate family members		3	10	2	0	0	4.07	0.15
e460 Societal attitudes		3	7	5	0	0	3.87	0.19
e575 General social support services, systems, and policies		4	10	0	1	0	4.13	0.18
e580 Health services, systems, and policies		4	7	2	2	0	3.87	0.26
e590 Labor and employment services, systems, and policies		4	7	4	0	0	4.00	0.19

Note: *indicates that the item did not fulfill the criteria; ^b^ indicates that the mean importance of items is between 3.0 to 3.5

(Corresponding to the second round of survey in the text)

### Personal factors

Personal factors are the special background of an individual’s life and survival, which are composed of personal characteristics that are not health conditions or health states. Personal factors have not been classified by ICF because of the large number of social and cultural differences. However, it may have an impact on the health and health-related conditions of individuals with health problems and we also explored the personal factors section while conducting two rounds of expert consensus. In the first round of the survey, the experts selected or proposed the personal factors related to women’s postpartum function, and in the second round, the importance of the items related to the personal factors was scored. A total of 8 individual factor items were identified with coefficients of variation ranging from 0.14 to 0.32. Detailed data are shown in Table 4.

**Table 4:** Personal factor survey data (*n* = 15)

Personal factors	The frequency of each score was evaluated for importance(persons)					Mean value	Coefficient of variation
	5	4	3	2	1		
Age	5	7	2	1	0	4.07	0.22
Educational background	4	5	4	2	0	3.73	0.28
Economical background	6	8	1	0	0	4.33	0.14
Occupational background	4	6	4	1	0	3.87	0.24
Position in the family	5	7	3	0	0	4.13	0.18
Personal attitudes	5	8	1	1	0	4.13	0.20
Lifestyle	6	7	2	0	0	4.27	0.16
Habits	5	3	4	3	0	3.67	0.32

## Discussion

A total of 69 items were preliminarily established in this study, including 32 body function items, 12 body structure items, 17 activity and participation items, 8 environmental factors items, and 8 personal factors items. This study is the first stage of exploring the construction of a postpartum functional assessment tool for women based on ICF in China and attempts to initially construct a category of postpartum functional assessment for women from the perspective of medical staff. Eventually ICF covers all four components of ICF and 21 field and does not contain personal factors in the ICF. These are also the most important impairments and limitations for postpartum women, as they may greatly affect the outcome of postpartum physical therapy interventions. In addition, they may provide some basis for helping postpartum women to develop the best rehabilitation treatment strategy.

In the dimension of body function, b28014 “Upper extremity pain”, b415 “Blood vessel functions”, and b430 “Blood system function” were deleted in the first round, which involved fewer abnormalities in clinical practice, and vascular function was difficult to be evaluate in clinical practice. Women in the postnatal often can appear a series of physical discomfort, in addition to the common pelvic dysfunction such as incontinence, sexual dysfunction, and pelvic organ prolapse, there will be some mental function obstacle problems such as sleep, emotion and memory function [3–5, 24], and these disorders may be due to biological factors, the reliance of the baby to care and acceptable level of social support.

In the dimension of body structure, s410 “Structure of the cardiovascular system”, s730 “Structure of the upper limbs”, and s7302 “Structure of the hand” was deleted in the first round, which were less abnormal at postpartum visit, and the “Structure of the cardiovascular system” was difficult to detect clinically. Body structure disorders are mainly reflected in s6 “Structures related to the urinary and reproductive system” [25], Impairment of pelvic floor muscle function is highly prevalent and may result from reduced tension, structural overload of the pelvic region, and trauma from fertility and childbirth, which is also our clinical focus at present.

In the dimension of activities and participation, due to postpartum sleep disorders, fatigue, pain, and other reasons [26], the daily activities such as “stress control”, “long-distance walking”, “doing housework” and “helping others” [27] are limited. These disorders may produce affective dysfunction such as anxiety, depression, and ultimately lead to some negative changes at the level of activity and participation.

In the dimension of environmental factors, e115 “Products and technology for personal use in daily living”, e120 “Products and technology for personal indoor and outdoor mobility and transportation”, e155 “Design, construction, and building products and technology of buildings for private use”, e315 “Extended family”, e325 “Acquaintances, peers, colleagues, neighbors, and community members”, e360 “Other professionals”, e540 “Transportation services, systems, and policies”, e5800 “Health services” were deleted in the first round. Among them, e115, e120, e155, and e540 were rarely involved in postpartum women. e315, e325, e360 experts explained that the reasons affecting women’s postpartum recovery were more the support and help of immediate family members [28]. e5800 had a large overlap with the retained item dimension. According to the current model of perinatal health promotion, the attitudes of family members, friends and health professionals play a crucial role as a support network for postpartum women, thus demanding that we must consider it from a biopsychosocial perspective.

Bulhões et al. [17]used the Delphi method to determine the ICF core set for women, including 15 body function components, 12 body structure components, 11 activity and participation components, 15 environmental factors components and 9 personal factors components. Compared with Brazil’s research, this research in terms of body function involves more mental functions, in addition to the common function of “emotional” and “sleep” function, this research also involves the “energy to drive” and “memory”; In terms of body structure in addition to the total of urogenital structures, Brazil consensus also involves “upper limb function”, etc.; Our country has the traditional custom of “sitting on the month”, and in terms of activities and participation, daily life activities are mainly restricted. However, in terms of activities and participation, Brazil has a lot of entries about interpersonal relationships. Brazil may pay more attention to the influence of environmental factors on people, and its environmental factors have more categories. These differences may be influenced by different cultural backgrounds.

In addition, the authority index of experts was 0.789 > 0.7 indicating that the authority of this study was good. After two rounds of expert consensus, the opinions of experts tended to be unified. In the second round of the survey, the coefficient of variation of item d415 “Maintaining a body position” was 0.31, and the coefficients of variation of other items were all < 0.3, which can be considered that the experts have a high degree of consistency and a high degree of recognition for ICF items in the assessment tool and reflect the good content validity of this study.

Due to individual differences, each woman’s postpartum impairment is different, that is, different functional items. Even if the functional items are the same, the degree of impairment is different and the degree of recovery of functional items is different. In addition, individual clinical recommendations must also be modified around the overall condition of the patient to achieve individualization. To cope with this complex functional state and to maximize the understanding of the impact of childbirth on women, the ICF system can provide a standardized terminology system, a unified evaluation framework, and comprehensive target management [29], so that we can better formulate rehabilitation goals for postpartum women and compare before and after treatment. The ICF-based postpartum function assessment tool has good content validity, which can provide a certain reference for clinical postpartum examination and assessment. Different from the traditional biomedical model, the ICF-based postpartum function assessment tool established by this expert consensus is based on the biological-psychological-social model [30]. Based on function, the comprehensive and maximum consideration of the impact of childbirth on women’s bodies and minds is conducive to the improvement of women’s postpartum quality of life and the prevention of some occult diseases.

At the same time, this study also has some shortcomings. First, although the experts selected in this study have rich clinical experience and professional level, more than half of them are from the postpartum rehabilitation department, and most of them are postpartum rehabilitation doctors and a few therapists. Secondly, this study only tested the content validity through the Delphi method and has not yet completed the reliability test. In the future, the assessment tool needs to be further verified through multi-center and large-sample clinical investigation to gradually improve.

## Conclusion

This study found that women have certain problems with their body function, structure, activity and participation during the postnatal period. The preliminary clinical investigation of this study is a single-center survey, and a multi-center survey can be carried out in the future to improve the representativeness of the sample. In addition, this study failed to conduct clinical application and effect tests for the formed categories of postpartum function assessment for women, and researchers need to conduct further research to understand the scientificity and practicability of this evaluation category and consider it for use in the electronic evaluation system. At the same time, the investigation process can immediately identify women’s physical and psychological problems in the postnatal period, reduce the probability of women developing related functional disorders in the postnatal period, and provide a comprehensive set of assessment tools for postnatal home visits, which can help to identify some hidden problems and provide immediate intervention to improve women’s health level.

### Electronic supplementary material

Below is the link to the electronic supplementary material.

Supplementary Material 1

## Data Availability

The datasets used and/or analyzed during the current study are available from the first or corresponding author upon reasonable request.

## Abbreviations

ICF: International Classification of Functioning, Disability and Health
WHO: World Health Organization
ICD: International Classification of Diseases
b: Body function
s: Body structure
d: Activity and participation
e: Environmental factors
Cr: Expert authority
Ca: Judgment coefficient
Cs: Degree of familiarity
CV: Coefficient of variation
σ: Standard deviations
M: Average
