# Supplementary material for: A postpartum functional assessment tool for women based on the international classification of functioning, disability and health
Source: BMC Womens Health. 2024 Jan 6;24:27. doi: 10.1186/s12905-024-02880-z (PMC10771640; doi:10.1186/s12905-024-02880-z)
Supplement: Supplementary file 1 — Supplementary Material 1 [file 12905_2024_2880_MOESM1_ESM.docx]

Additional file

**Supplementary Table 1 (**83 items)

| ICF category | very important | important | moderately important | not very important | not important |
| --- | --- | --- | --- | --- | --- |
| b1300 Energy functions |  |  |  |  |  |
| b134 Sleep functions |  |  |  |  |  |
| b144 Memory functions |  |  |  |  |  |
| b152 Emotional functions |  |  |  |  |  |
| b280 Sensation of pain |  |  |  |  |  |
| b28011 Chest pain |  |  |  |  |  |
| b28012 Stomach and abdomen pain |  |  |  |  |  |
| b28013 Back pain |  |  |  |  |  |
| b28014 Upper extremity pain |  |  |  |  |  |
| b28015 Lower extremity pain |  |  |  |  |  |
| b2802 Pain in multiple parts of the body |  |  |  |  |  |
| b415 Blood vessel functions |  |  |  |  |  |
| b420 Blood pressure function |  |  |  |  |  |
| b430 Blood system function |  |  |  |  |  |
| b440 Respiration functions |  |  |  |  |  |
| b4552 Easy fatigue |  |  |  |  |  |
| b460 Sensations related to cardiovascular and respiratory function |  |  |  |  |  |
| b515 Digestive functions |  |  |  |  |  |
| b525 Defecation functions |  |  |  |  |  |
| b535 Sensations associated with the digestive system |  |  |  |  |  |
| b555 Endocrine gland function |  |  |  |  |  |
| b620 Urination functions |  |  |  |  |  |
| b6202 Urinary continence |  |  |  |  |  |
| b640 Sexual functions |  |  |  |  |  |
| b660 Reproductive functions |  |  |  |  |  |
| b6603 Lactation |  |  |  |  |  |
| b670 Sensations related to reproduction and reproductive function |  |  |  |  |  |
| b730 Muscle power functions |  |  |  |  |  |
| b7305 Power of muscles of the trunk |  |  |  |  |  |
| b735 Muscle tone functions |  |  |  |  |  |
| b740 Muscular endurance function |  |  |  |  |  |
| b760 Control of voluntary movement functions |  |  |  |  |  |
| b770 Gait |  |  |  |  |  |
| b780 Sensations related to muscle and motor function |  |  |  |  |  |
| b820 Repair functions of the skin |  |  |  |  |  |
| s410 Structure of cardiovascular system |  |  |  |  |  |
| s430 Structure of respiratory system |  |  |  |  |  |
| s610 Structure of urinary system |  |  |  |  |  |
| s620 Structure of pelvic floor |  |  |  |  |  |
| s630 Structure of reproductive system |  |  |  |  |  |
| s6300 Ovary |  |  |  |  |  |
| s6301 Structure of uterus |  |  |  |  |  |
| s6302 Breast and nipple |  |  |  |  |  |
| s6303 Structure of vagina and external genitalia |  |  |  |  |  |
| s730 Structure of upper extremity |  |  |  |  |  |
| s7302 Structure of hand |  |  |  |  |  |
| s740 Structure of pelvic region |  |  |  |  |  |
| s750 Structure of lower extremity |  |  |  |  |  |
| s760 Structure of trunk |  |  |  |  |  |
| s7601 Muscles of trunk |  |  |  |  |  |
| d230 Carrying out daily routine |  |  |  |  |  |
| d240 Handling stress and other psychological demands |  |  |  |  |  |
| d415 Maintaining a body position |  |  |  |  |  |
| d450 Walking |  |  |  |  |  |
| d570 Looking after one’s health |  |  |  |  |  |
| d5700 Ensuring one’s physical comfort |  |  |  |  |  |
| d5701 Control diet and regulate body |  |  |  |  |  |
| d640 Doing housework |  |  |  |  |  |
| d660 Assisting others |  |  |  |  |  |
| d6600 Assisting others with self-care |  |  |  |  |  |
| d760 Family relationships |  |  |  |  |  |
| d770 Intimate relationships |  |  |  |  |  |
| d7702 Sexual relationships |  |  |  |  |  |
| d850 Remunerative employment |  |  |  |  |  |
| d920 Recreation and leisure |  |  |  |  |  |
| d9201 Sports |  |  |  |  |  |
| d9205 Socializing |  |  |  |  |  |
| e115 Products and technology for personal use in daily living |  |  |  |  |  |
| e120 Products and technology for personal indoor and outdoor mobility and transportation |  |  |  |  |  |
| e155 Design, construction, and building products and technology of buildings for private use |  |  |  |  |  |
| e310 Immediate family |  |  |  |  |  |
| e315 Extended family |  |  |  |  |  |
| e320 Friends |  |  |  |  |  |
| e325 Acquaintances, peers, colleagues, neighbors, and community members |  |  |  |  |  |
| e355 Health professionals |  |  |  |  |  |
| e360 Other professionals |  |  |  |  |  |
| e410 Individual attitudes of immediate family members |  |  |  |  |  |
| e460 Societal attitudes |  |  |  |  |  |
| e540 Transportation services, systems, and policies |  |  |  |  |  |
| e575 General social support services, systems, and policies |  |  |  |  |  |
| e580 Health services, systems, and policies |  |  |  |  |  |
| e5800 Health services |  |  |  |  |  |
| e590 Labor and employment services, systems, and policies |  |  |  |  |  |

**Supplementary Table 2** Items and frequencies of "body function, body structure, activity and participation, environmental factors" in the ICF core category of postpartum functioning of women

| ICF category | Frequency of patient disorders (%) | Medical staff investigated the frequency of disorders (%) |
| --- | --- | --- |
| b1300 Energy functions | 73.3 | 96.7 |
| b134 Sleep functions | 68.3 | 96.7 |
| b144 Memory functions | 55.7 | 83.3 |
| b152 Emotional functions | 68.3 | 100.0 |
| b280 Sensation of pain | 73.7 | 100.0 |
| b28011 Chest pain | 31.3 | 80.0 |
| b28012 Stomach and abdomen pain | 30.3 | 76.7 |
| b28013 Back pain | 41.0 | 86.7 |
| b2802 Pain in multiple parts of the body | 45.0 | 30.0 |
| b420 Blood pressure function | 3.7 | 53.3 |
| b430 Blood system function | 43.3 | 63.3 |
| b4552 Easy fatigue | 68.3 | 100 |
| b515 Digestive functions | 9.7 | 66.7 |
| b525 Defecation functions | 60.3 | 100 |
| b535 Sensations associated with the digestive system | 9.0 | 66.7 |
| b555 Endocrine gland function | 11.0 | 56.7 |
| b620 Urination functions | 37.0 | 100.0 |
| b6202 Urinary continence | 33.0 | 100.0 |
| b640 Sexual functions | 57.0 | 100.0 |
| b660 Reproductive functions | 58.3 | 90.0 |
| b6603 Lactation | 50.0 | 90.0 |
| b670 Sensations related to reproduction and reproductive function | 38.0 | 93.3 |
| b730 Muscle power functions | 85.7 | 100.0 |
| b735 Muscle tone functions | 75.0 | 100.0 |
| b740 Muscular endurance function | 88.0 | 100.0 |
| b780 Sensations related to muscle and motor function | 40.3 | 66.7 |
| b820 Repair functions of the skin | 64.3 | 96.7 |
| s610 Structure of urinary system | 97.3 | 100.0 |
| s620 Structure of pelvic floor | 88.0 | 100.0 |
| s630 Structure of reproductive system | 86.3 | 100.0 |
| s6301 Structure of uterus | 32.3 | 100.0 |
| s6302 Breast and nipple | 30.7 | 100.0 |
| s6303 Structure of vagina and external genitalia | 65.7 | 100.0 |
| s7302 Structure of hand | 27.0 | 63.3 |
| s740 Structure of pelvic region | 82.7 | 100.0 |
| s760 Structure of trunk | 91.0 | 100.0 |
| s7601 Muscles of trunk | 90.0 | 100.0 |
| d230 Carrying out daily routine | 56.3 | 80.0 |
| d240 Handling stress and other psychological demands | 64.0 | 96.7 |
| d415 Maintaining a body position | 38.3 | 60.0 |
| d450 Walking | 70.3 | 56.7 |
| d570 Looking after one’s health | 82.0 | 90.0 |
| d5700 Ensuring one’s physical comfort | 73.7 | 83.3 |
| d5701 Control diet and regulate body | 39.3 | 80.0 |
| d640 Doing housework | 50.7 | 83.3 |
| d660 Assisting others | 44.3 | 56.7 |
| d6600 Assisting others with self-care | 44.3 | 53.3 |
| d7702 Sexual relationships | 36.0 | 86.7 |
| d850 Remunerative employment | 27.0 | 90.0 |
| d920 Recreation and leisure | 83.3 | 93.3 |
| d9201 Sports | 76.7 | 80.0 |
| d9205 Socializing | 55.0 | 83.3 |
| e115 Products and technology for personal use in daily living | 60.0 | 76.7 |
| e310 Immediate family | 99.0 | 100.0 |
| e315 Extended family | 48.0 | 80.0 |
| e320 Friends | 85.3 | 86.7 |
| e325 Acquaintances, peers, colleagues, neighbors, and community members | 44.0 | 83.3 |
| e355 Health professionals | 71.3 | 93.3 |
| e360 Other professionals | 75.0 | 53.3 |
| e410 Individual attitudes of immediate family members | 99.0 | 100.0 |
| e575 General social support services, systems, and policies | 65.7 | 90.0 |
| e580 Health services, systems, and policies | 92.3 | 100.0 |
| e590 Labor and employment services, systems, and policies | 26.7 | 86.7 |

Note: Example standards for integration are as follows

| ICF category | Frequency of patient disorders (%) | Medical staff investigated the frequency of disorders (%) | Final inclusion result |
| --- | --- | --- | --- |
| b1300 Energy functions | ≥30% | ＞50% | Inclusion |
| b28014 Upper extremity pain | ＜30% | ≤50% | exclusion |
| b2802 Pain in multiple parts of the body | ≥30% | ≤50% | Inclusion |
| b420 Blood pressure function | <30% | ＞50% | Inclusion |
